# Association between Dry Eye Parameters Depends on Tear Components

**DOI:** 10.3390/jcm11113056

**Published:** 2022-05-28

**Authors:** Shu-Wen Chang, Wan-Lin Wu

**Affiliations:** 1 Department of Ophthalmology, Far Eastern Memorial Hospital, New Taipei City 220, Taiwan; arielwu10101@gmail.com; 2 Department of Ophthalmology, National Taiwan University Hospital, Taipei 100, Taiwan

**Keywords:** superficial punctate keratitis, dry eye parameters, expressible meibomian glands, dry eye pathophysiology, blink/partial blink rates

## Abstract

How tear components contribute to dry-eye symptoms/signs remains less well-defined. This observational cross-sectional study enrolled 4817 (F/M = 3590/1227) patients. Subjective symptoms were evaluated with the SPEED and OSDI questionnaires. Fluorescein tear breakup time (FTBUT), superficial punctate keratitis (SPK) grading, Schirmer scores, number of expressible meibomian glands (MGE), lipid layer thickness (LLT), blink/partial blink rates and meibography were recorded. Patients were divided into 4 types according to their Schirmer scores and LLT, i.e., Type 1 (N = 1494): Schirmer > 5 mm, LLT > 60 nm; Type 2 (N = 698): Schirmer > 5 mm, LLT ≤ 60 nm; Type 3 (N = 1160): Schirmer ≤ 5 mm, LLT ≤ 60 nm; Type 4 (N = 1465): Schirmer ≤ 5 mm, LLT > 60 nm. Lipid deficiency (LLT ≤ 60 nm) and aqueous deficiency (Schirmer score ≤ 5 mm) were found in 38.6% and 54.5% of patients, respectively. The majority (62.4%) of lipid-deficient patients were also aqueous deficient, while 44.2% of aqueous-deficient patients were also lipid-deficient. Type 3 patients (mixed type) had the highest symptom scores (*p* = 0.008 and 0.007 for SPEED and OSDI, respectively), more total blinks (*p* < 0.001) and the shortest FTBUT (*p* < 0.001). Stepwise multiple regression demonstrated that LLT and Schirmer score were significant contributors to FTBUT in all 4 types. The FTBUT correlated with SPK severity in all 4 types, with Schirmer score in types 1 and 4, and with LLT in type 3 patients. SPK correlated with LLT and MGE in types 1 and 4. Age correlated with dry eye parameters more significantly than sex. Subtyping by aqueous and lipid components facilitates the understanding of dry eye pathophysiology.

## 1. Introduction

Dry eye (DE) disease has a prevalence of 5% to 50%, with a higher prevalence in women than in men [1,2]. The accurate diagnosis and classification of DE is challenging owing to the wide variations in symptoms and the lack of a single reliable clinical assessment. Via morphological meibomian gland (MG) evaluation, population-based studies indicated that up to 69% of patients with DE exhibit anatomic abnormalities in the MG [3], while a clinic-based cohort study showed that 85.5% of patients with DE exhibited signs of MG dysfunction (MGD) [4]. However, meibography alone cannot discriminate MGD from non-MGD [5]. In addition, associations between DE signs and symptoms are low and inconsistent, with a correlation coefficient between −0.4 and 0.4 in most studies [6]. Subclassification of DE as predominantly evaporative or aqueous-deficient has been widely implemented [7]. A fluorescein tear film breakup time (FTBUT) of ≤5 s and the presence of subjective dry eye symptoms are used for the diagnosis of DE in Japan and some beyond [8,9,10,11]. A new concept of “tear-film-oriented diagnosis” using a tear film breakup pattern was proposed for the differential diagnosis and treatment of dry eye disease (DED), which includes aqueous deficiency dry eye, decreased wettability dry eye, and increased evaporation dry eye [8,9,11,12,13]. This approach is conceptually ideal and makes “tear-film-oriented therapy” easily acceptable to both ophthalmologists and patients [13]. However, there is a barrier to execution, as it relies largely on the subjective classification of tear film breakup pattern recognition by the experience of ophthalmologists. Further studies to increase understanding of the pathogenesis of DE and to find the reliable and relevant measures of disease are needed to enhance clinical assessment of DE and the measurement of response to therapeutic interventions.

Most of the total tear volume consists of the aqueous layer, while the tear film lipid layer accounts for only 2–3% [14]. Both aqueous and lipid amounts are correlated with FTBUT but to different degrees [15,16]. The FTBUT was shorter in patients with MGD than in those without MGD [16]. In addition to the commonly adopted lid margin and meibography evaluation of MG, quantification of lipid layer thickness (LLT) with or without interferometer instruments has become an important technology in the evaluation of MG function and treatment effects [17,18,19,20,21,22,23,24]. Subjective symptoms are one of the major diagnostic criteria in dry eyes. The Ocular Surface Disease Index (OSDI) and Standard Patient Evaluation of Eye Dryness Questionnaire (SPEED) are two commonly used questionnaires to quantify subjective symptoms, with moderate association [7,17,25]. However, it is difficult to distinguish between MGD and DE on the basis of symptoms alone [26]. Moreover, the association between symptoms and signs varies among studies [27]. How the major tear components, i.e., aqueous and lipid tear amounts, contribute to subjective symptoms and objective signs remains less well-defined. In this study, we subtyped dry eye patients by the two major tear components and analyzed the relationship among dry eye parameters according to the most commonly measured tear components, i.e., aqueous tears and LLTs.

## 2. Materials and Methods

### 2.1. Patients

This study protocol was approved by the Institutional Review Board at Far Eastern Memorial Hospital and was conducted in accordance with the Declaration of Helsinki. We retrospectively reviewed the examination results of 4913 patients visiting the dry eye clinic of a senior ophthalmologist for dry eye management between August 2015 and December 2021.

**Figure 1:**
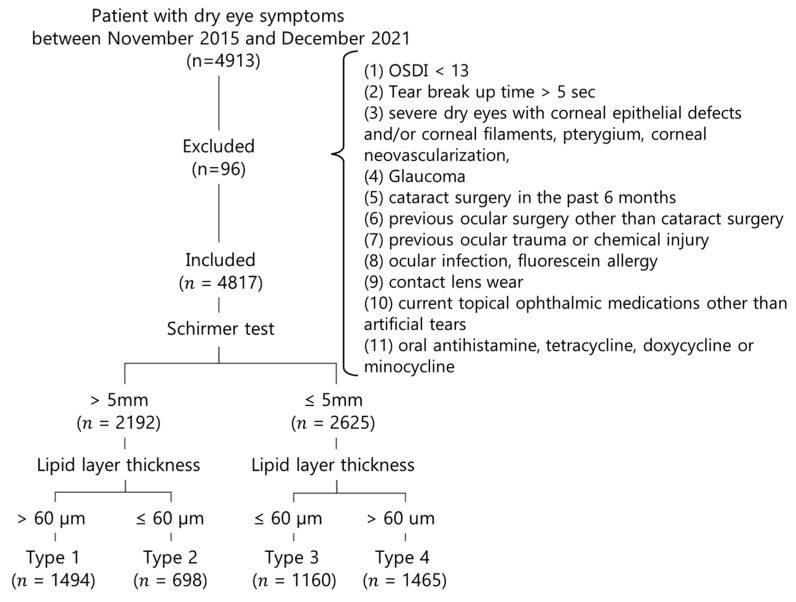
Patient selection flowchart.

We included patients whose Ocular Surface Disease Index (OSDI) score was ≥13, whose FTBUT was ≤5 s and who were at least 20 years of age (Figure 1). The exclusion criteria were patients with insignificant symptoms of OSDI <13, severe dry eyes with corneal epithelial defects and/or corneal filaments, pterygium, corneal neovascularization, glaucoma, previous ocular surgery (other than cataract surgery), active ocular trauma and ocular infection, fluorescein allergy, contact lens wear, current topical medication use (other than artificial tears) and oral antihistamine, tetracycline, doxycycline or minocycline. Cases with an FTBUT > 5 s were also excluded. There were 4817 cases eligible for analysis after excluding 96 cases.

Patients were divided into 4 types according to their Schirmer scores and LLT as follows. Type 1 (N = 1494): Schirmer > 5 mm, LLT > 60 nm; Type 2 (N = 698): Schirmer > 5 mm, LLT ≤ 60 nm; Type 3 (N = 1160): Schirmer ≤ 5 mm, LLT ≤ 60 nm; Type 4 (N = 1465): Schirmer ≤ 5 mm, LLT > 60 nm. Data in the right eyes were included for analysis.

### 2.2. Study Protocol

Patients who used artificial tears, gels or ointments were instructed not to apply them for at least 12 h before examination. They were instructed not to wear periocular cosmetics on the day of examination. In our dry eye center, all participants first completed two questionnaires for subjective symptoms, i.e., Standardized Patient Evaluation of Eye Dryness (SPEED) and OSDI. They then underwent lipid layer thickness (LLT), blink/partial blink rate measurement and meibography examination with the LipiView^®^ II interferometer (Johnson & Johnson Vision). Subsequently, FTBUT and SPK were recorded by an ophthalmologist. After that, the numbers of expressible meibomian glands (MGEs) were counted, and meibomian gland loss was graded as meiboscale by one well-trained examiner based on meibography images captured by LipiView^®^ II. Finally, aqueous tear secretion was evaluated by the Schirmer test with topical anesthetics measured at 5 min using standard 35 × 5 mm tear test strips (Eagle Vision, Katena Products, Parsippany-Troy Hills, NJ, USA).

### 2.3. Subjective Symptoms

We included the frequency, severity and total scores of the SPEED questionnaire [28] as well as the total OSDI score and the three subtotal scores of the OSDI questionnaire (frequency of symptoms, frequency of activity limitation and frequency of environmental factors triggering discomfort) [7,29,30] for further analysis.

### 2.4. Lipid Layer Thickness (LLT)

LLT was measured with a LipiView II^®^ interferometer. The LLT was presented in interferometric color units, in which 1 interferometric color unit corresponds to approximately 1 nm [31]. The average LLT was recorded. The upper limit of LLT detected by LipiView II^®^ was 100 nm, and values greater than 100 nm were recorded as 100+ nm and were coded 110 for calculation as previously reported [17,25]. The numbers of total and partial blinks during the 20 s of examination were also recorded for further analysis.

### 2.5. Number of Expressible Meibomian Glands (MGEs)

The expressibility of meibomian secretion was evaluated with a handheld Meibomian Gland Evaluator™ applied to the nasal, central, and temporal regions of both the upper and lower eyelids as previously described [17,25]. The MGE was counted under a slit-lamp biomicroscope. The MGE of the upper lid, lower lid and sum of both eyelids was used for analysis.

### 2.6. Meiboscale

The meibomian gland images were captured by a LipiView II^®^ interferometer. The meibomian gland dropout was graded as meiboscale, ranging from degree 0 to 4 (degree 0, no gland loss; degree 1, ≤25% area of gland loss; degree 2, 26–50% area of gland loss; degree 3, 51–75% area of gland loss; degree 4, >75% area of gland loss) [32]. The meiboscale of the upper eyelid and lower eyelid and the average of both lids were used for analysis.

### 2.7. Blink Patterns

The number of total/partial blinks in 20 s was measured by a LipiView^®^ II interferometer. The number of total blinks was the sum of partial and complete blinks. The partial blink rate was the number of partial blinks ÷ number of total blinks × 100% [17,25].

### 2.8. Fluorescein Tear-Film Breakup Time (FTBUT) and Superficial Punctate Keratitis (SPK)

The FTBUT was measured (average of three) after applying fluorescein solution onto the bulbar conjunctiva [7,15]. The corneal/conjunctival staining patterns were graded by an ophthalmologist from 0–4 according to the Oxford scheme [33].

### 2.9. Statistical Analysis

All statistical analyses were performed using SPSS v.20.0 (IBM SPSS Statistics for Windows, IBM Corp., Armonk, NY, USA). All numeric variables were assessed for normality using the Kolmogorov–Smirnov test. The Kruskal–Wallis test and post hoc analysis with Dunn’s test were used to compare the numeric variables, including the SPEED questionnaire score, OSDI questionnaire score, LLT, Schirmer test results, meiboscale grades, number of MGEs, and total/partial blinks, among the dry eye subtypes. Descriptive results are presented as the mean ± standard deviation (SD). Spearman’s rank correlation coefficient was used to determine the correlations between relevant parameters. Stepwise multiple regression analysis was used to select appropriate parameters and to build a regression model for explaining the relationship between the FTBUT and the chosen parameters separately in the 4 types. As age and sex are two well-recognized factors in dry eyes [2,29,30], the correlation between age/sex and the subjective and objective parameters in the 4 subtypes was also analyzed. FTBUT is one of the most important measurable diagnostic parameters in dry eye disease [12,13]; thus, we conducted multiple linear regression analysis to delineate its determinant parameters in the 4 subtypes. The chi-square test was used to examine the sex distribution; *p* values of <0.05 were considered statistically significant.

## 3. Results

There were 4817 patients with an age of 54.8 ± 15.0 years old. The 4817 enrolled patients had an SPEED score of 11.4 ± 5.5 and an OSDI score of 38.4 ± 22.4. This indicates that most of them had moderate-to-severe dry eyes. Our included patients had a Schirmer score of 5.7 ± 5.4 mm, an LLT of 70.6 ± 24.3 nm and an FTBUT of 2.9 ± 1.2 s. After subtyping the patients using a cutoff LLT of 60 nm and a cutoff value of Schirmer score of 5 mm, 38.6% (1858/4817) of them were lipid-deficient (≤60 nm), including 698 in type 2 (14.5%, pure lipid-deficient) and 1160 in type 3 (24.1%, mixed type). In contrast, 54.5% (2625/4817) of patients were aqueous-deficient, including those in type 3 (34.5%, 1660/4817 mixed type) and type 4 (30.4%, 1465/4817 pure aqueous-deficient).

**Figure 2:**
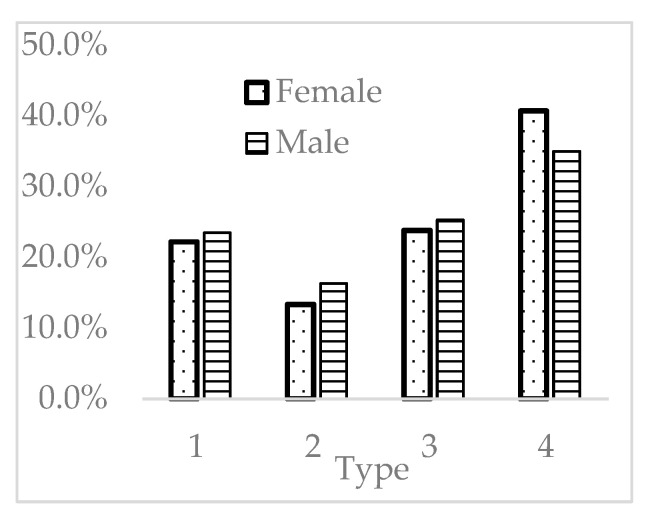
Distribution of females and males in the 4 types.

Females accounted for 74.5% (3590/4817) of the entire group, compatible with previous understanding that females are a major risk factor for dry eyes. In addition, females had significantly fewer type 2 and more type 4 patients (*p* = 0.002) (Figure 2).

### 3.1. Difference among Dry Eye Types

#### 3.1.1. Age and Sex

There was a significant difference in age among the 4 subtypes (*p* < 0.001). Types 2 and 3 patients were significantly younger, while those in type 4 were the oldest (Table 1).

#### 3.1.2. Symptom Scores

There was a significant difference in the symptoms evaluated with both SPEED and OSDI among the 4 types (Table 1). There was a significant difference in symptom scores among groups (*p* = 0.008 and 0.007 for SPEED and OSDI, respectively). Type 3 patients had significantly higher SPEED scores than type 1 patients (*p* = 0.001), especially in the severity score. Type 3 and 4 patients had significantly higher OSDI scores (*p* = 0.007), especially the environmental triggering factor (*p* = 0.004). There were 61.9% (2981/4817) of the patients in the severe symptoms (OSDI > 33) group, although there was no significant difference in the distribution of mild (OSDI 13–22), moderate (OSDI 23–32), and severe symptoms (OSDI > 33) among the 4 types (*p*= 0.294) (Table 2).

#### 3.1.3. Lipid-Related Parameters

Types 1 and 4 patients had significantly thicker LLTs than those in types 2 and 3 (*p* < 0.001) (Table 3), who also had fewer secreting glands, i.e., MGE, than those in types 1 and 4 (*p* < 0.001). The MG loss was more severe in the upper lids than in the lower lids. A total of 46.1% and 22.2% of patients had a meiboscale grade of >1 of the upper lid and lower lid, respectively. However, there was no significant difference in the meiboscale among the 4 types (*p* = 0.861).

#### 3.1.4. Aqueous Secretion and Number of Blinks

Type 3 and 4 patients had lower Schirmer scores than type 1 and 2 patients (*p* < 0.001) (Table 3). Types 2 and 3 patients, whose LLT was <60 nm, had more total blinks (*p* < 0.001) (Table 3). There was no difference in the number of partial blinks among the 4 subtypes. The partial blink rate was lower in types 2 and 3 than in types 1 and 4 (*p* < 0.001).

#### 3.1.5. Tear Film Stability

The entire group had an FTBUT of 2.9 ± 1.2 s. Type 1 patients had a significantly longer FTBUT than all the other 3 types (*p* < 0.001) (Table 3).

#### 3.1.6. Superficial Punctate Keratitis (SPK)

Type 2 patients had lower SPK grades than all the other 3 types (*p* = 0.032) (Table 3).

### 3.2. Correlations among Parameters

#### 3.2.1. Symptom Scores

Age correlated with subjective symptoms and lipid/aqueous-associated parameters more significantly than sex did in all 4 groups (Table 4). The association between age and SPEED/OSDI was more noticeable in types 1 and 4 (Table 4). Age correlated negatively with SPEED frequency and severity scores in types 1, 3 and 4 but not in type 2 patients. In contrast, age positively correlated OSDI frequency scores in types 1 and 4 but negatively with environment triggering scores in types 1, 3 and 4.

#### 3.2.2. Lipid-Related Parameters

Age was associated positively with LLT and negatively with MGE of both upper and lower lids (Table 4) but only significantly in types 1 and 4 patients. In contrast, age correlated positively with meiboscale grade. The correlation was significant in the upper eyelids of all 4 types of patients, while it was significant in the lower eyelids only in types 1 and 4 patients.

#### 3.2.3. Aqueous Secretion and Number of Blinks

Age correlated negatively with Schirmer scores in types 1 and 2 but not in types 3 and 4 (Table 4). Sex was not associated with most of the examined parameters except LLT. Age correlated negatively with the number of total blinks in types 1, 3 and 4 (Table 4) and the number of partial blinks in all types (Table 4).

#### 3.2.4. SPK Grades

SPK severity correlated positively with age in type 1 (Table 4) and negatively with FTBUTs in all 4 types (Table 5). It correlated positively with LLT and negatively with MGE in types 1 and 4 (Table 5). However, SPK did not correlate with the Schirmer score in any of the 4 types. The negative association between SPK and MGE was significant in groups 1 and 4, whose LLT was thicker than 60 nm.

#### 3.2.5. Tear Film Stability

Age also correlated negatively with FTBUT but was significant only in type 1 patients (Table 4). FTBUT correlated with Schirmer scores in types 1 and 4 (Table 6), whose LLT was >60 nm. In contrast, the FTBUT correlated positively with the LLT in type 3 patients (Table 6).

### 3.3. Stepwise Multiple Linear Regression Model

Table 7 summarizes the variables involved in the final equation of stepwise regression at the 0.05 level in each type. LLT and Schirmer score were significant contributors to FTBUT in all 4 types. Age was significant in types 2, 3, and 4, while sex was significant in types 1, 3 and 4. MGE of the upper lid was significant in types 3 and 4, while MGE of the lower lid was significant only in type 4. In contrast, meiboscale was significant only in type 1.

## 4. Discussion

Most of the dry eye diseases encountered in daily life involve short FTBUT-type dry eye [13]. The two currently measurable major components of tear film are lipid layer thickness by interferometer and aqueous secretion by Schirmer test/anterior segment optical coherent tomography. To facilitate the understanding of the association among dry eye parameters, we classified our dry eye patients according to these two components and conducted association analysis accordingly. The secretory tear mucin and membrane-associated mucins that contribute to the reduced wettability are currently immeasurable clinically and thus are not adopted into the classification criteria. We excluded patients with FTBUT > 5 s because of the relatively small number of cases in our patient pool. Among the 4913 retrospectively reviewed patients, 4817 (98.0%) had an FTBUT of ≤5 s. The Asia Dry Eye Society (ADES) consensus has declared that a Schirmer score of less than 5 mm in 5 min is indicative of ADDE [12]. We thus used a Schirmer score of 5 mm as the cutoff value. For a cutoff value of ≤60 nm LLT measured by LipiView, the sensitivity for the detection of an MGD was 47.9%, and the specificity was 90.2% [31]. We used the cutoff value of 60 nm for dry eye subtyping in this study. Our included patients with significant subjective symptoms and shortened FTBUT fully fulfilled the diagnosis of dry eyes per Japan/ADES criteria [8,9,12]. A total of 61.9% of the 4817 patients had severe symptoms (OSDI ≥ 33), indicating that our patients were relatively symptomatic.

After subtyping the patients using a cutoff LLT of 60 nm and a cutoff value of Schirmer score of 5 mm, 38.6% of them were lipid-deficient (≤60 nm) while 54.5% of patients were aqueous-deficient. The majority of lipid-deficient patients (24.1%/38.6% = 62.4%) also had an aqueous deficient component. Similarly, a substantial proportion of aqueous-deficient dry eye patients (24.1%/54.5% = 44.2%) were also lipid-deficient, having an LLT of ≤60 nm. This is in concordance with a previous report that 43.4% of dry eye patients exhibited lipid deficiency, and 56.6% of them exhibited aqueous deficiency using dynamic interferometry [27]. Similar to Ji et al., we also advocate that conventional assessments should be combined with interferometric tear analysis to determine the most appropriate treatment for each dry eye patient.

The FTBUT is a major diagnostic parameter according to the ADES. Both aqueous and lipid components contribute to FTBUT, but to different degrees [15,16]. Using stepwise multiple linear regression analysis, we confirmed that LLT and Schirmer score were two major determinants of FTBUT in all 4 types of dry eyes (Table 7). The FTBUT correlated with Schirmer scores in types 1 and 4 (Table 6), whose LLT was >60 nm. This confirms that the amount of aqueous tears contributes significantly to FTBUT [15], particularly in the presence of a sufficient protective LLT of >60 nm. Consequently, patients with more aqueous tears had longer FTBUTs. In contrast, the FTBUT correlated positively with the LLT in type 3 patients (Table 6). This implies that an LLT of 60 nm is essential in the protection of tear evaporation-related shortening of the FTBUT [15]. In contrast, an LLT of ≤60 nm is insufficient to provide adequate evaporation protection. The aqueous tear evaporation-related thinning of the tear film and thus shortening of the FTBUT is dependent on the LLT thickness when it is ≤60 nm [15]. This is in agreement with a previous study demonstrating a shorter FTBUT in ADDE patients with MGD than in those without MGD [16].

Deficiency in either aqueous or lipid components leads to subnormal tear film stability. Type 1 patients comprised 31.0% (1494/4817) of our included patients. They had the longest FTBUT, as they had both sufficient aqueous and lipid tears. However, having an FTBUT of 3.2 ± 1.5 s, type 1 patients still suffer from dry eye symptoms, having an OSDI sores of 37.3 ± 21.7. They were neither aqueous-deficient nor lipid-deficient. We thus suggest that their short FTBUT could have resulted from “mucin deficiency” or “decreased wettability” according to the definition of the ADES [8,9,12].

The SPK severity correlated negatively with FTBUT in all types. This is compatible with previous study that a short FTBUT is potentially associated with SPK [34]. In addition, the SPK severity correlated negatively with MGE in types 1 and 4, affirming the protective effects of actively secreting MG on the ocular surface integrity. Paradoxically, the SPK severity also correlated positively with LLT in types 1 and 4. Since the SPK severity also correlated with age, we suggest that the abnormally thick LLT measured in elderly patients with severe dermatochalasis could have contained denatured meibum and sebum, which were potentially damaging to the corneal epithelium [25].

Types 2 and 3 patients had a thinner LLT and fewer MGE than those in types 1 and 4. They also had higher SPEED scores and more TB than those in types 1 and 4. This implies that the lipid layer could play significant roles in the protection and reflex blinking and SPEED symptoms. In contrast, types 3 and 4 had higher OSDI scores and lower Schirmer scores than types 1 and 2, affirming the contribution of aqueous tears to the OSDI scores [35].

Yoshikawa et al. reported that the severity of eye pain is greater in aqueous-deficient dry eye and decreased wettability dry eye than in increased evaporative dry eye [33]. In our study, both types 3 and 4 were aqueous-deficient, while types 2 and 3 were increased evaporative dry eyes. It is thus reasonable that type 3 and 4 patients had the highest OSDI scores in our study.

Although age and sex are two well-recognized factors in dry eyes [2,29,30], age was more influential than sex in this study. We illustrated that age was positively associated with LLT and negatively associated with MGE in types 1 and 4 patients (Table 4), whose LLT was >60 nm. Similarly, we also found that old age was associated with low Schirmer scores, which was significant only in type 1 and 2 patients whose Schirmer scores were >5 mm (Table 4). This is reasonable, as the association between parameters can be better delineated only when there is a large difference between the maximum and minimum readings. A small difference between the maximum and minimum makes readings almost constant, and thus, no association could be found. These correlations could have been concealed if subtyping was not considered.

Dry eye is defined as “a multifactorial disease characterized by unstable tear film” according to the ADES definition [13], while it is defined as “a multifactorial disease of the ocular surface” in the Tear Film and Ocular Surface Society (TFOS DEWSII) definition [34]. In this study, patients with types 2, 3, and 4 had tear film abnormalities and short FTBUTs, while those with type 1 might have abnormal ocular surfaces, i.e., decreased wettability and related short FTBUT dry eyes. Our method of subtyping short FTBUT links the two definitions and makes the understanding and potential treatment option more easily comprehensible to the patients.

The strength of this study is that we included a relatively large number of patients with short FTBUTs visiting the same ophthalmologist. Interobserver variation was thus eliminated. Subclassifying dry eye patients using tear film components makes pathophysiologic analysis more easily understandable. Our results support the concept of tear-film-oriented diagnosis [8,9,11,12,13] and facilitate tear-film-oriented treatment for dry eye [8].

One limitation of our study is that we included only patients with an FTBUT of ≤5 s for subtyping analysis. Our conclusion might not be generalizable to patients with an FTBUT >5 s. As patients visiting tertiary hospitals for dry eye treatment would possibly represent more severe cases, a future general eye clinic-based study could facilitate the understanding of the subtype distribution. Another limitation is that LLT is subjected to short-term fluctuation clinically, e.g., cataract surgery [35] and lid hygiene [25]. Periocular sebum can be measured if the interferometric measurement is not conducted appropriately. Standardized preexamination instruction is preferred for more accurate measurement to facilitate better MG evaluation.

## 5. Conclusions

The association between dry eye parameters depends on tear components. Subtyping by aqueous and lipid components facilitates the understanding of dry eye pathophysiology.

## Figures and Tables

**Table 1:** Differences in age and symptom questionnaires among dry eye subtypes.

	Type 1 (1494)	Type 2 (698)	Type 3 (1160)	Type 4 (1465)	Total (4817)	*p*
Age (years)	55.5 ± 15.1 ^#,§,+^	51.4 ± 15.8 *^,+^	52.3 ± 14.4 *^,+^	57.7 ± 14.2 *^,#,§^	54.8 ± 15.0	**<0.001**
SPEED	11.1 ± 5.4 ^§^	11.5 ± 5.6	11.8 ± 5.6 *	11.4 ± 5.6	11.4 ± 5.5	**0.008**
Frequency (SPEED)	5.2 ± 2.5 ^§^	5.3 ± 2.5	5.5 ± 2.6 *	5.3 ± 2.6	5.3 ± 2.5	**0.029**
Severity (SPEED)	5.9 ± 3.2 ^#,§,+^	6.2 ± 3.3 *	6.3 ± 3.2 *	6.1 ± 3.4 *	6.1 ± 3.3	**0.008**
OSDI	37.3 ± 21.7 ^§,+^	37.0 ± 21.9 ^§^	39.8 ± 23.1 *^,#^	39.2 ± 22.5 *	38.4 ± 22.4	**0.007**
OSDI (Frequency)	7.7 ± 4.5	7.4 ± 4.4 ^§,+^	7.9 ± 4.5 ^#^	8.0 ± 4.6 ^#^	7.8 ± 4.5	**0.029**
OSDI (Activity limitation)	4.5 ± 3.7 ^§^	4.7 ± 3.8	5.0 ± 3.9 *^,+^	4.6 ± 3.8 ^§^	4.7 ± 3.8	**0.014**
OSDI (Environment)	3.8 ± 3.4 ^§^	3.8 ± 3.5 ^§^	4.2 ± 3.7 *^,#^	4.0 ± 3.6	3.9 ± 3.5	**0.004**

Numbers in parenthesis represent case number in the indicated type. *: *p* < 0.05 as compared with type 1; ^#^: *p* < 0.05 as compared with type 2; ^§^: *p* < 0.05 as compared with type 3; ^+^: *p* < 0.05 as compared with type 4.

**Table 2:** Case distribution of OSDI severity among subtypes.

	OSDI Score			
	13–22	23–32	>33	Total
Type 1	274 (18.4%)	299 (20.0%)	920 (61.6%)	1494 (100.0%)
Type 2	150 (21.4%)	145 (20.7%)	404 (57.8%)	698 (100.0%)
Type 3	220 (19.0%)	218 (18.8%)	722 (62.3%)	1160 (100.0%)
Type 4	256 (17.5%)	274 (18.7%)	935 (63.8%)	1465 (100.0%)
Total	900 (18.7%)	936 (19.4%)	2981 (61.9%)	4817 (100.0%)

Numbers in parentheses represent percentages within the OSDI range of the indicated dry eye subtype. Chi-square *p* = 0.294.

**Table 3:** Summary of objective dry eye parameters.

	Type 1 (1494)	Type 2 (698)	Type 3 (1160)	Type 4 (1465)	Total (4817)	*p*
**Lipid-associated parameters**						
LLT (nm)	79.5 ± 17.5 ^#,§,+^	46.9 ± 9.7 *^,+^	46.8 ± 10.1 *^,+^	91.8 ± 15.8 *^,#,§^	70.6 ± 24.3	**<0.001**
MGE	8.2 ± 4.2 ^#,§,+^	7.3 ± 3.9 *^,+^	7.7 ± 4.1 *^,+^	8.8 ± 4.4 *^,#,§^	8.1 ± 4.2	**<0.001**
MGE (upper)	4.6 ± 2.9 ^#,+^	4.2 ± 2.8 *^,§,+^	4.5 ± 2.9 ^#,§,+^	5.1 ± 3.0 *^,#,§^	4.7 ± 2.9	**<0.001**
MGE (lower)	3.6 ± 2.3 ^#,§^	3.1 ± 2.2 *^,+^	3.2 ± 2.4 *^,+^	3.6 ± 2.3 ^#,§^	3.4 ± 2.3	**<0.001**
Meiboscale (grade)	1.4 ± 0.7	1.4 ± 0.8	1.4 ± 0.7	1.4 ± 0.7	1.4 ± 0.7	0.861
Meiboscale (upper)	1.6 ± 0.9	1.6 ± 0.9	1.6 ± 0.9	1.6 ± 0.9	1.6 ± 0.9	0.303
Meiboscale (lower)	1.3 ± 0.7	1.3 ± 0.8	1.3 ± 0.7	1.2 ± 0.7	1.3 ± 0.7	0.146
**Aqueous secretion, blink patterns, tear film stability, and corneal staining**						
Schirmer (mm)	7.9 ± 5.8 ^#^^,^^§,^^+^	11.2 ± 6.2 *^,^^§,^^+^	2.8 ± 1.6 *^,^^#,^^+^	3.3 ± 3.1 *^,^^#,^^§^	5.7 ± 5.4	**<0.001**
TB	7.2 ± 5.0 ^#^^,^^§^	8.0 ± 5.0 *^,^^+^	8.0 ± 5.1 *^,+^	7.1 ± 5.0 ^#^^,^^§^	7.5 ± 5.0	**<0.001**
PB	4.4 ± 4.0 ^#^^,^^§^	4.1 ± 3.7 *	4.1 ± 3.6 *	4.3 ± 4.1	4.2 ± 3.9	0.099
PB (%)	60.9 ± 34.5 ^#^^,^^§^	53.7 ± 35.5 *^,+^	54.2 ± 34.0 *^,^^+^	61.2 ± 34.6 ^#^^,^^§^	58.3 ± 34.7	**<0.001**
TBUT (sec)	3.2 ± 1.5 ^#^^,^^§,^^+^	2.9 ± 1.2 *	2.7 ± 1.0 *	2.9 ± 1.2 *	2.9 ± 1.2	**<0.001**
SPK (grade)	0.3 ± 0.5 ^#^	0.2 ± 0.6 *^,§,+^	0.3 ± 0.6 ^#^	0.3 ± 0.6 ^#^	0.3 ± 0.6	0. 032

MGE: number of expressible meibomian gland expression; PB: number of partial blinks; TB: number of total blinks; PB (%): partial blink rate; FTBUT: fluorescein tear-film break-up time; SPK: superficial punctate keratitis; Numbers in parenthesis represent case number in the indicated type. *: *p* < 0.05 as compared with type 1; ^#^: *p* < 0.05 as compared with type 2; ^§^: *p* < 0.05 as compared with type 3; ^+^: *p* < 0.05 as compared with type 4.

**Table 4:** Correlation of age and sex with subjective symptoms and lipid-associated parameters.

Correlations		Age				Sex			
		Type 1	Type 2	Type 3	Type 4	Type 1	Type 2	Type3	Type 4
**Symptom scores**									
SPEED	r_s_=	−0.153	0.146	−0.238	−0.094	−0.137	0.003	−0.004	−0.045
	*p*=	**0.001**	0.108	**<0.001**	**0.034**	**0.002**	0.975	0.948	0.313
SPEED (Frequency)	r_s_=	−0.140	0.168	−0.191	−0.080	−0.138	−0.033	−0.018	−0.084
	*p*=	**0.002**	0.064	**0.001**	0.070	**0.002**	0.717	0.746	0.059
SPEED (Severity)	r_s_=	−0.146	0.120	−0.253	−0.094	−0.120	0.031	0.008	−0.012
	*p*=	**0.001**	0.187	**<0.001**	**0.034**	**0.007**	0.731	0.882	0.796
OSDI	r_s_=	0.045	0.106	−0.035	0.088	−0.217	−0.090	−0.084	−0.126
	*p*=	0.317	0.244	0.535	**0.049**	**<0.001**	0.324	0.136	**0.004**
OSDI (Frequency)	r_s_=	0.115	0.165	0.008	0.158	−0.192	−0.046	−0.094	−0.160
	*p*=	**0.010**	0.067	0.886	**<0.001**	**<0.001**	0.612	0.094	**<0.001**
OSDI (Activity limitation)	r_s_=	−0.088	0.028	−0.095	−0.062	−0.073	0.056	0.071	0.026
	*p*=	**0.049**	0.761	0.093	0.162	0.104	0.538	0.206	0.558
OSDI (Environment)	r_s_=	−0.149	−0.014	−0.133	−0.111	−0.169	−0.178	−0.085	−0.100
	*p*=	**0.001**	0.878	**0.018**	**0.012**	**<0.001**	**0.049**	0.131	**0.025**
**Lipid-associated parameters**									
LLT	r_s_=	0.126	−0.004	−0.016	0.173	−0.107	−0.152	0.120	−0.091
	*p*=	**0.005**	0.964	0.784	**<0.001**	**0.017**	0.093	**0.034**	**0.041**
MGE	r_s_=	−0.136	−0.044	−0.087	−0.167	−0.011	−0.047	0.021	−0.032
	*p*=	**0.002**	0.632	0.121	**<0.001**	0.806	0.605	0.712	0.468
MGE (upper)	r_s_=	−0.107	−0.093	−0.050	−0.148	−0.095	−0.022	0.028	−0.078
	*p*=	**0.017**	0.305	0.378	**0.001**	**0.034**	0.810	0.622	0.078
MGE (lower)	r_s_=	−0.109	0.033	−0.089	−0.122	0.087	−0.056	0.005	0.031
	*p*=	**0.015**	0.715	0.115	**0.006**	0.053	0.535	0.932	0.485
meiboscale	r_s_=	0.263	0.295	0.194	0.275	−0.001	0.083	−0.074	−0.041
	*p*=	**<0.001**	**0.001**	**0.001**	**<0.001**	0.983	0.368	0.199	0.363
Meiboscale (upper)	r_s_=	0.301	0.380	0.266	0.312	0.025	0.052	−0.067	−0.018
	*p*=	**<0.001**	**<0.001**	**<0.001**	**<0.001**	0.582	0.567	0.238	0.694
Meiboscale (lower)	r_s_=	0.155	0.145	0.057	0.151	−0.020	0.085	−0.083	−0.034
	*p*=	**0.001**	0.110	0.316	**0.001**	0.652	0.350	0.140	0.449
**Blink and other tear parameters**									
Schirmer	r_s_=	−0.138	−0.268	0.011	−0.034	−0.002	0.195	−0.076	0.027
	*p*=	**0.002**	**0.003**	0.840	0.438	0.962	**0.031**	0.179	0.539
TB	r_s_=	−0.191	−0.171	−0.186	−0.127	−0.035	−0.001	−0.095	−0.125
	*p*=	**<0.001**	0.058	**0.001**	**0.004**	0.442	0.993	0.091	**0.005**
PB	r_s_=	−0.212	−0.234	−0.142	−0.169	−0.070	−0.150	−0.037	−0.077
	*p*=	**<0.001**	**0.009**	**0.011**	**<0.001**	0.117	0.098	0.512	0.084
PB rate (%)	r_s_=	−0.176	−0.124	−0.024	−0.134	−0.026	−0.046	0.024	−0.012
	*p*=	**<0.001**	0.175	0.672	**0.003**	0.568	0.613	0.667	0.786
FTBUT	r_s_=	−0.208	−0.007	−0.030	−0.043	0.100	0.020	0.044	0.021
	*p*=	**<0.001**	0.937	0.592	0.333	**0.026**	0.824	0.433	0.631
SPK	r_s_=	0.134	0.048	−0.027	−0.001	−0.080	0.103	−0.128	−0.130
	*p*=	**0.003**	0.602	0.634	0.981	0.077	0.263	**0.024**	**0.003**

LLT: average lipid-layer thickness; MGE: number of expressible meibomian glands; TB: number of total blinks; PB: number of partial blinks; PB (%): partial blink rate; FTBUT: fluorescein tear-film break-up time; SPK: superficial punctate keratitis; *p*: statistically significant by Spearman’s rank correlation.

**Table 5:** Correlations between SPK and lipid/aqueous parameters.

	Type 1		Type 2		Type 3		Type 4	
	r_s_	*p*	r_s_	*p*	r_s_	*p*	r_s_	*p*
FTBUT	−0.245	**<0.001**	−0.186	**0.042**	−0.243	**<0.001**	−0.177	**<0.001**
LLT	0.095	**0.034**	0.022	0.813	−0.061	0.279	0.174	**<0.001**
MGE	−0.122	**0.006**	−0.036	0.697	−0.067	0.239	−0.117	**0.009**
meiboscale	0.086	0.059	0.212	**0.022**	0.100	0.085	0.104	**0.022**
Schirmer	−0.055	0.220	−0.079	0.388	−0.027	0.629	−0.081	0.069

**Table 6:** Correlations between FTBUT and lipid/aqueous parameters.

	Type 1		Type 2		Type 3		Type 4	
	r_s_	*p*	r_s_	*p*	r_s_	*p*	r_s_	*p*
LLT (nm)	0.020	0.662	−0.010	0.908	0.112	**0.047**	−0.007	0.867
MGE	0.057	0.205	−0.232	**0.010**	0.085	0.131	0.070	0.115
MGE (upper)	0.058	0.195	−0.266	**0.003**	0.033	0.558	0.019	0.666
MGE (lower)	0.031	0.492	−0.093	0.306	0.102	0.069	0.098	**0.027**
Meiboscale (grade)	−0.042	0.353	0.075	0.415	−0.096	0.096	−0.080	0.075
Meiboscale (upper) (grade)	−0.048	0.288	0.025	0.780	−0.076	0.177	−0.049	0.267
Meiboscale (lower) (grade)	−0.021	0.644	0.085	0.349	−0.089	0.113	−0.096	**0.031**
Schirmer (mm)	0.107	**0.018**	0.018	0.847	0.098	0.084	0.110	**0.013**

LLT: average lipid-layer thickness; MGE: number of expressible meibomian glands; TB: number of total blinks; PB: number of partial blinks; PB (%): partial blink rate; FTBUT: fluorescein tear-film break-up time; SPK: superficial punctate keratitis; *p*: statistically significant by Spearman’s rank correlation.

**Table 7:** Summary of stepwise multiple linear regression models for FTBUT.

	Type 1		Type 2		Type 3		Type 4		Type 1–4	
	Beta	*p*	Beta	*p*	Beta	*p*	Beta	*p*	Beta	*p*
LLT (nm)	0.600	**<0.001**	0.616	**<0.001**	0.572	**<0.001**	0.383	**<0.001**	0.328	**<0.001**
Schirmer score (mm)	0.182	**<0.001**	0.140	**0.008**	0.110	**<0.001**	0.142	**<0.001**	0.117	**<0.001**
Age (Y)			0.189	**0.019**	0.167	**0.001**	0.194	**<0.001**	0.237	**<0.001**
MGE (upper)					0.102	**0.002**	0.133	**<0.001**	0.127	**<0.001**
MGE (lower)							0.083	**0.001**	0.070	**<0.001**
Sex	0.059	**0.012**			0.038	**0.041**	0.057	**<0.001**	0.057	**<0.001**
Meiboscale (grades)	0.121	**0.004**							0.083	**<0.001**
Adjusted R^2^	0.810		0.838		0.874		0.830		0.826	

LLT: average lipid-layer thickness; MGE: number of expressible meibomian glands; TB: number of total blinks; PB: number of partial blinks; PB (%): partial blink rate; FTBUT: fluorescein tear-film break-up time; SPK: superficial punctate keratitis; Beta: standardized coefficients; *p*: statistical significance by stepwise multiple regression analysis.

## Data Availability

The data presented in this study are available on request from the corresponding author. The data are not publicly available because the Ethical Review Board has not approved the public availability of these data.
